# Loss of oral mucosal stem cell markers in oral submucous fibrosis and their reactivation in malignant transformation

**DOI:** 10.1038/s41368-020-00090-5

**Published:** 2020-08-21

**Authors:** Mohit Sharma, Felipe Paiva Fonseca, Keith D. Hunter, Raghu Radhakrishnan

**Affiliations:** 1 Department of Oral Pathology, Sudha Rustagi College of Dental Sciences and Research, Faridabad, Haryana India; 2 Department of Oral Surgery and Pathology, School of Dentistry, Federal University of Minas Gerais, Belo Horizonte, MG Brazil; 3 Academic Unit of Oral and Maxillofacial Medicine and Pathology, School of Clinical Dentistry, University of Sheffield, Claremont Crescent, Sheffield, UK; 4 Department of Oral Pathology, Manipal College of Dental Sciences, Manipal Academy of Higher Education, Manipal, Karnataka India

**Keywords:** Cancer stem cells, Oral cancer detection

## Abstract

The integrity of the basal stem cell layer is critical for epithelial homoeostasis. In this paper, we review the expression of oral mucosal stem cell markers (OM-SCMs) in oral submucous fibrosis (OSF), oral potentially malignant disorders (OPMDs) and oral squamous cell carcinoma (OSCC) to understand the role of basal cells in potentiating cancer stem cell behaviour in OSF. While the loss of basal cell clonogenicity triggers epithelial atrophy in OSF, the transition of the epithelium from atrophic to hyperplastic and eventually neoplastic involves the reactivation of basal stemness. The vacillating expression patterns of OM-SCMs confirm the role of keratins 5, 14, 19, CD44, β1-integrin, p63, sex-determining region Y box (SOX2), octamer-binding transcription factor 4 (Oct-4), c-MYC, B-cell-specific Moloney murine leukaemia virus integration site 1 (Bmi-1) and aldehyde dehydrogenase 1 (ALDH1) in OSF, OPMDs and OSCC. The downregulation of OM-SCMs in the atrophic epithelium of OSF and their upregulation during malignant transformation are illustrated with relevant literature in this review.

## Introduction

The basal stem cell layer of normal oral mucosa (NOM) is a self-perpetuating reservoir of cells with a mechanism for self-renewal, a property referred to as clonogenicity or stemness. The integrity of the basal stem cell layer is thus essential for epithelial homoeostasis. Breakdown in cell-cycle turnover is antecedent to the development of oral potentially malignant disorders (OPMDs) and oral squamous cell carcinoma (OSCC). Oral submucous fibrosis (OSF) is an OPMD commonly present among people in the Indian subcontinent and Southeast Asia.^1,2^ Various epidemiological studies implicate areca nut chewing as the main aetiological factor in OSF. There is overwhelming evidence suggesting that the chewing of commercial addictive products, such as pan masala, gutka, mawa and betel quid (BQ) containing considerable amounts of areca nut, tobacco and slaked lime, predisposes patients to OSF.^1,3^ Areca nut has cytotoxic effects on oral mucosal cells,^4^ and disturbingly, oral cancer arising in the background of OSF seems to develop earlier and has a greater propensity to invade and metastasize.^1^

**Fig. 1:**
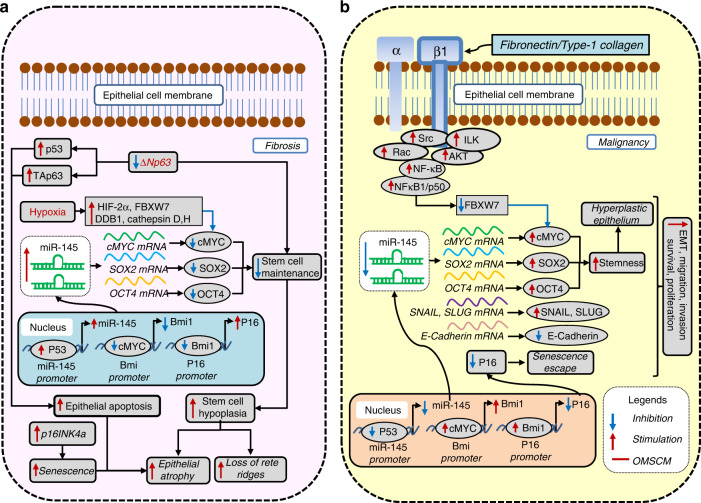
c-MYC, SOX2 and OCT-4 as oral mucosal stem cell markers (OM-SCMs) in oral submucous fibrosis (OSF). **a** Their downregulation mediates epithelial atrophy, and **b** their upregulation mediates malignancy

**Fig. 2:**
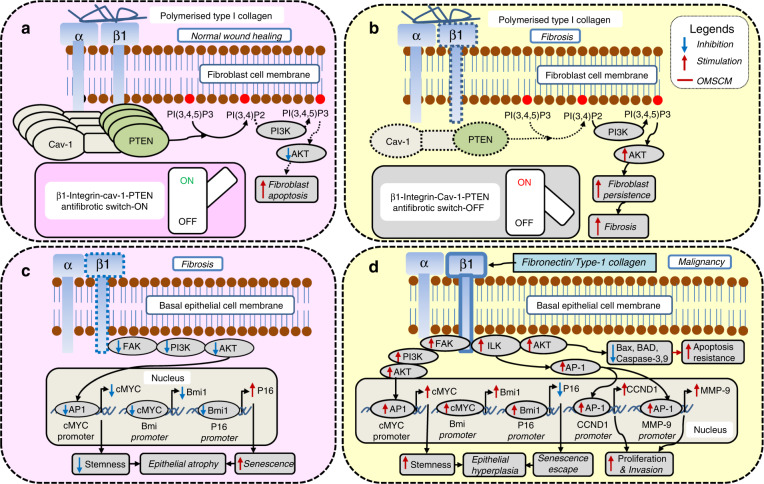
β_1_-integrin as an oral mucosal stem cell marker (OM-SCM) in oral submucous fibrosis (OSF). **a** In fibroblasts, normal β_1_-integrin levels are antifibrotic, and **b** reduced β_1_-integrin levels promote fibrosis. In epithelial cells, **c** decreased β_1_-integrin levels promote epithelial atrophy, and **d** increased β1-integrin levels promote malignancy

**Fig. 3:**
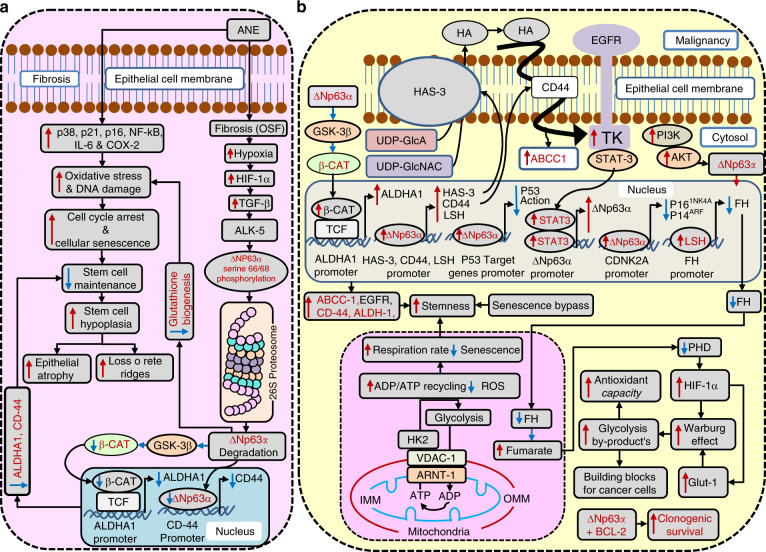
∆Np63α as an oral mucosal stem cell marker (OM-SCM) in oral submucous fibrosis (OSF). **a** Its downregulation mediates epithelial atrophy, and **b** its upregulation mediates malignancy

**Fig. 4:**
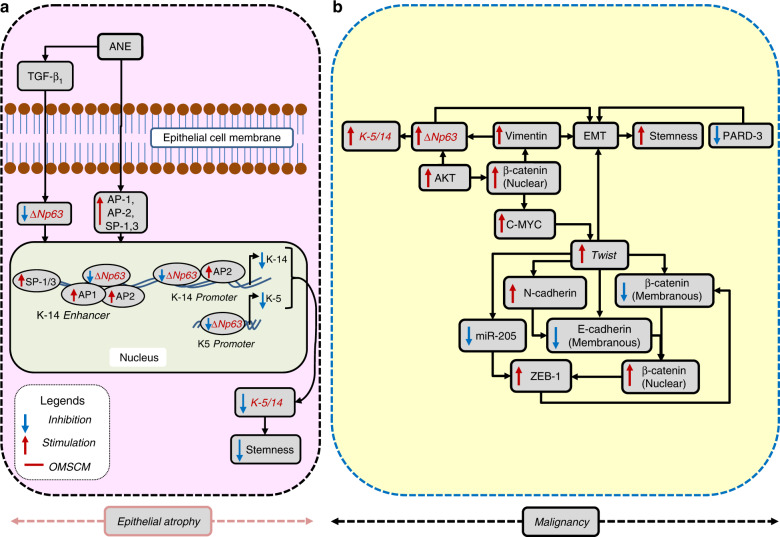
K-5/14 as an oral mucosal stem cell marker (OM-SCM) in oral submucous fibrosis (OSF). **a** Its downregulation mediates epithelial atrophy, and **b** its upregulation mediates malignancy

**Fig. 5:**
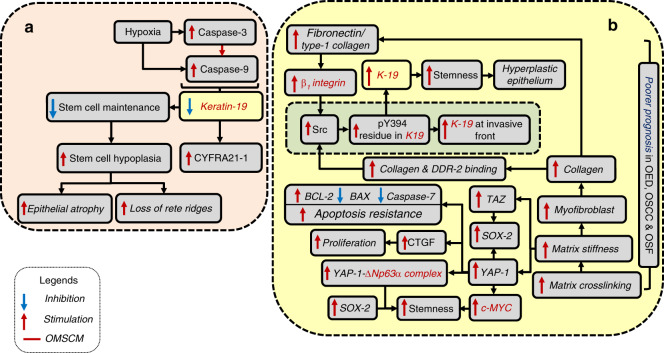
K-19 as an oral mucosal stem cell marker (OM-SCM) in oral submucous fibrosis (OSF). **a** Its downregulation mediates epithelial atrophy, and **b** its upregulation mediates malignancy

Considering OSF as an over-healing wound, the role of stem cell activity in its genesis is well documented.^2,5,6^ Several reports suggest downregulated basal stem cell activity as a tipping event triggering epithelial atrophy in OSF.^4,7–14^ Limited scientific evidence supports a rebound amplification of stem cell activity in the epithelium transitioning from atrophic OSF to oral epithelial dysplasia (OED) and eventually to OSCC. A comprehensive assessment of oral mucosal stem cell markers (OM-SCMs) in relation to the progression of OSF, OED and OSCC is performed in this review (Figs. 1–5).

## Stemness regulation: the role of wild-type versus mutated p53

When mutated, p53 triggers a cascade of events leading to malignancy. However, its role in OSF and its malignant transformation are not clear. Since p53 antibodies (e.g., p53-duo) do not distinguish between wild-type p53 (Wt-p53) and mutated p53 (Mut-p53), it is critical to delineate their role in the progression of OSF.

Wt-p53 expression seems to be vital for the initiation of fibrosis to the extent that the expression of profibrotic plasminogen activator inhibitor-1 (PAI-1) is re-established following the expression of Wt-p53.^15^ Transforming growth factor-beta (TGF-β) induces the complex formation between Wt-p53 and Smads2/3/4 in the PAI-1 promoter, recruiting the histone acetyltransferase CREB-binding protein (CBP). CBP augments histone H3 acetylation in the PAI-1 promoter, activating PAI-1 transcription.^16^ Thus, Wt-p53 is expressed intensely in the basal layer of the atrophic epithelium in OSF compared to the hyperplastic epithelium,^13^ suggesting that Wt-p53 plays a key role in the initiation of fibrosis and epithelial atrophy by reducing stemness.

Wt-p53 represses stemness by inducing miR-145,^17^ which exerts tumour-suppressor functions through the downregulation of c-MYC, octamer-binding transcription factor 4 (Oct-4) and sex-determining region Y-box 2 (SOX2).^17,18^ Notably, atrophic epithelium in OSF shows high p53 levels, low c-MYC expression and stable hypoxia-inducible factor (HIF) expression.^13^ The clonal expansion and evolution of dysplasia is the outcome of high c-MYC activity and Mut-p53 expression.^13^ The downregulation of Oct-4 in the atrophic epithelium of OSF in contrast to the normal epithelium^4^ (Fig. 1a) and its rebound expression in the malignant transformation of OSF suggests altered stemness (Fig. 1b).^19^ Thus, it could be concluded that Wt-p53 works as an anti-stemness factor and is associated with fibrosis and atrophy, while Mut-p53 is associated with dysplasia and malignant progression.^20^

## Alterations in the expression pattern of OM-SCMs in OSF, OPMD and OSCC

The OM-SCs in the basal layer of the oral mucosa are the normal stem cells essential for maintaining the integrity of the oral mucosa.^21^ Contact with the basement membrane is required to maintain basal keratinocyte stemness. The severity of the contact of OM-SCs with the basement membrane promotes their differentiation.^22^ Their biological attributes, such as inherent longevity and the ability to self-replicate, make these cells an ideal candidate to accumulate a full complement of mutations triggering tumorigenesis. Hence, their breakdown is reflected in the aberrant expression of OM-SCMs in various OPMDs and OSCC. Although the stem cells associated with cancer are quite similar to normal stem cells and express the same markers, certain unique characteristics, such as the loss of growth control, justify their separate designation as cancer stem cells (CSCs) or tumour-initiating cells.^21,23,24^

Several studies have shown that CSCs play a crucial role in the growth, spread and recurrence of oral cancer.^25–31^ CSCs in oral cancer demonstrate elevated expression levels of stem cell markers, such as Oct-4, SOX2, Nanog, Nestin, CK19, B-cell-specific Moloney murine leukaemia virus integration site 1 (Bmi-1), CD117 (c-kit), CD44 and CD133, and decreased expression levels of involucrin and CK-13.^25,26,32–34^ Evidence supporting this stems from the finding that the signalling pathways regulating normal stem cell division (i.e., Notch, Wnt, Hedgehog and Bmi-1) are involved in oral cancer development.^27,30,31,35,36^ Recent evidence indicates that CSCs within surgical margins play a crucial role in the clinical outcomes of cancer.^37–39^ Lazarevic et al.^38^ showed that the surgical margins of oral cancer express stem cell markers, such as CD44, Oct-4, CD133, Nanog and SOX2, which have the ability to form spheroids to become resistant to chemotherapy. CSCs survive and promote cancer, as they maintain low levels of reactive oxygen species (ROS) levels and exhibit redox patterns matching those of normal stem cells.^23,40^ This explains their resistance to elimination by radiotherapy.^23,40^

Xu et al.^41^ showed that enhanced aerobic glycolysis and l-lactate production in oral CSCs is mediated via the epidermal growth factor (EGF)/epidermal growth factor receptor (EGFR)/phosphoinositide 3-kinase (PI3K)/HIF-1α pathway, and is evidenced by the upregulation of the CD44^+^CD24^−^ population of CSCs along with the expression of other CSC markers, such as Bmi-1 and aldehyde dehydrogenase 1 (ALDH1). Zhao et al.^42^ showed that the cancer stem cell-like state in oral cancer is primed by lactate uptake, and is evidenced by the expression of CD133 and upregulated Wnt signalling.

Individually, ALDH1 and CD44 are considered markers of CSCs in oral cancer,^31,43^ and together, they identify the specific CSC compartment.^43–46^ ALDH1 was found to be upregulated at the invasive tumour front (ITF), and the adjacent non-tumour epithelium correlated with tumour aggressiveness.^47^ Aldehyde dehydrogenase 1 family member A1 (ALDH1A1) may thus serve as a marker for premalignant cells in oral cancer.^48^ CD44^high^/ALDH1^high^ cells exhibit a greater tumour sphere-forming capability than CD44^high^/ALDH1^low^ cells.^46^ Remarkably, vimentin-positive spindle-shaped cells were found in the CD44^high^/ALDH1^high^ cell population but not in the CD44^high^/ALDH1^low^ cell population,^46^ indicating the propensity of the former to undergo epithelial–mesenchymal transition (EMT) and seed new tumours. Certainly, the increased expression of vimentin correlates with higher migratory activity and oral cancer progression.^39^

Other stemness markers, such as CD44, CD133, glucose-regulated protein 78 (Grp-78), Grp-96, Oct-4, Nanog and SOX2, are upregulated upon exposure to areca nut and correlate with worse prognosis in areca nut-induced cancers.^49,50^ Furthermore, areca nut-induced chemoradioresistance results from the upregulation of ATP-binding cassette subfamily G member-2 (ABCG-2), a drug- efflux pump and a stem cell marker.^22,49,50^ Oral cancers among habitual areca nut chewers demonstrate an aggressive phenotype, chemoradioresistance and a much lower 5-year survival rate than those without areca habits.^18^

Studies on oral premalignant lesions have shown that CSC markers, such as ABCG-2 and Bmi-1, predict the transformation of oral leukoplakia to cancer.^51^ The expression patterns of CSC markers, such as ALDH1 and CD133, correlate with a high risk of malignant transformation of oral leukoplakia.^52^ In addition, the coexpression of the CSC markers ALDH1 and Bmi-1 is a strong indicator of malignant transformation of oral erythroplakia.^20^ It is well established that the CSC markers ALDH1, Bmi-1 and ABCG-2 drive the process of field cancerization in oral erythroplakia.^53,54^

An intriguing correlation of stem cell activity in OSF, OPMD and OSCC was uncovered through our literature search. While Rajendran et al.^7^ was the first to propose downregulated stem cell activity in OSF, it was subsequently confirmed to be due to the adverse effects of nitric oxide and areca nut-associated carcinogens on basal stem cells.^11^ The reduced stem cell activity in atrophic OSF was evidenced by a decrease in the expression of proliferative/stem cell markers, such as Ki-67, Cyclin-D1 (CCND-1) and c-MYC,^10,13^ and an increase in their expression in the malignant transformation of OSF.^10,13^

The OM-SCMs involved in molecular signalling pathways include keratins 5/14, 15, 19, α_6_β_4_-integrin^+^CD71^−^, β_1_-integrin, collagen IV, p75^NGFR^, stage-specific embryonic antigen 1 (SSEA1), CD24, CD44 (CD44H), CD71, CD117 (c-kit), CD133, melanoma-associated chondroitin sulfate proteoglycan (MCSP), Nestin, p63, octamer-binding transcription factor-3/4 (Oct-3/4), Nanog, SOX2, ABCG-2, ALDH1 and Bmi-1 (Supplementary Information (SI)).^22,55–65^ The crucial role of keratins 5, 14, 19, CD44, β1-integrin, p63, SOX2, Oct-4, c-MYC, Bmi-1 and ALDH1 in potentiating stem cell behaviour in OSF is further discussed in sections “β1-integrin as a stem cell marker in OSF and its malignant evolution”, “p63 as a stem cell marker in OSF and its malignant transformation”, “c-MYC as a stem cell marker in OSF and its malignant evolution”, “Bmi-1 is a stem cell marker in OSF and its malignant evolution”, “Keratin 5/14 as a stem cell marker in OSF and its malignant evolution” and “Keratin-19 as a stem cell marker in OSF and its malignant evolution”.

### β1-integrin as a stem cell marker in OSF and its malignant evolution

β_1_-integrin functions as OM-SCM since it is downregulated in differentiated cells. The modulation of collagen synthesis during wound healing occurs via β_1_-integrin. Polymerized collagen inhibits an excessive accumulation of ECM by activating β1-integrin.^9,66^ When fibroblasts interact with polymerized type I collagen, caveolin-1 (Cav-1) forms a complex with phosphatase and tensin homologue (PTEN) and β1-integrin on the plasma membrane.^66^ This places PTEN in the correct spatial location to inhibit the PI3K/AKT signal generated through the β_1_-integrin–matrix interaction,^66^ thus promoting fibroblast apoptosis (Fig. 2a). Conversely, the downregulation of β1-integrin leads to reduced membrane accumulation of the PTEN–Cav-1–β_1_-integrin complex.^9,66^ This hampers the ability of PTEN to inactivate AKT signalling,^66^ leading to fibroblast persistence and promoting fibrosis (Fig. 2b).

Hyperplastic OSF and OSCC demonstrate increased stem cell activity, as evidenced by the upregulation of β_1_-integrin.^9^ An increased β1-integrin expression pattern correlates with a greater tumorigenic potential, as indicated by the augmented tumour spheres and holoclonal colony formation in oral cancer cells compared to control cells.^67^ The β_1_-integrin-driven transition from dormancy to tumorigenicity occurs through its activation by fibronectin/type-1 collagen and/or the activation of uPA receptor (uPAR).^68^ β1-integrin, through the integrin-linked kinase (ILK)/AKT pathway, inhibits various proapoptotic enzymes, such as BCL-2-associated X apoptosis regulator (BAX), BCL-2-associated agonist of cell death (BAD) and caspase-3/-9, and thereby promotes apoptosis resistance in cancer cells.^69^ Increased ILK also upregulates CCND-1, which promotes cellular proliferation, and matrix metalloproteinase-9 (MMP-9), promoting the invasive potential.^69^ β1-integrin also upregulates c-MYC through the focal adhesion kinase (FAK)/PI3K/AKT pathway,^69,70^ resulting in increased stemness and hyperplastic epithelium in OSF (Fig. 2d). Conversely, the downregulation of β1-integrin in the epithelial compartment should lead to an atrophic epithelium. Certainly, very advanced OSF with a severely atrophic epithelium does show the lowest stem cell activity, evidenced by the downregulation of β_1_-integrin in buccal mucosa (Fig. 2c).^9^

### p63 as a stem cell marker in OSF and its malignant transformation

The p63 gene products occur in six different protein isoforms, of which ∆Np63α, ∆Np63β and ∆Np63γ are devoid of the N-terminal transactivation domain, whereas TAp63α, TAp63β and TAp63γ act as transcription factors. Among these, ∆Np63α is the predominant isoform whose expression is reduced in OSF without OED and increased in OSF with severe OED.^12,14^ The expression of ∆Np63α confirms the regenerative potential, as it is restricted to the basal stem cell layer in NOM.^61^ Downregulated ∆Np63α mediates senescence,^61,71^ which is considered to be a barrier to tumour development. Oxidative stress and DNA damage, which mediate cell-cycle arrest and the senescence of keratinocytes in OSF following exposure to areca nut extract (ANE), are a result of the overactivity of p16, p21, p38, nuclear factor kappa B (NF-κB), IL-6 and COX-2 (Fig. 3a).^72^

∆Np63α functions as a dominant-negative inhibitor of p53, competing with its DNA-binding sites to promote oral cancer.^73^ Thus, the downregulation of ∆Np63α activates Wt-p53,^15^ further contributing to apoptosis and epithelial atrophy in OSF. Likewise, ∆Np63α inhibits TAp63, which mirrors p53 function.^12,74^ If ∆Np63α is reduced, the unhampered TAp63 mediates apoptosis and epithelial atrophy in OSF (Fig. 1a).

TGF-β_1_-TGFβR1 (ALK-5)-mediated serine-66/68 phosphorylation of the ∆Np63α isoform induces 26S proteasomal degradation (Fig. 3a).^61^ Impaired ∆Np63α isoform function through TGF-β_1_^61^ might be responsible for oxidative stress-mediated epithelial atrophy in OSF, as reported by Khan et al.^4^ and Wang et al.^75^ (Fig. 3a). Indeed, ∆Np63α has the ability to inhibit the cell death induced through oxidative stress, DNA damage and anoikis via the upregulation of glutathione biogenesis, and it cooperates with B-cell lymphoma 2 (BCL-2) to promote clonogenic survival (Fig. 3a, b).^75^ Since ∆Np63α is a positive regulator of OM-SCMs, ALDH1 and CD44, its downregulation^61,76^ is reflected in impaired basal stem regeneration (Fig. 3a).^61,76^ The decreased stem cell maintenance manifests as stem cell hypoplasia, epithelial atrophy and loss of rete ridges (Fig. 3a).

The augmentation of ∆Np63α in keratinocytes mediates senescence bypass through the chromatin- remodelling protein lymphoid-specific helicase (Lsh) and endows them with self-renewal abilities, indicated by the presence of a K-15^+^ stem cell population.^77^ In addition, ∆Np63α promotes the repression of p16^Ink4a^ and p19^ARF^, especially in the presence of retrovirus-associated DNA sequencing (RAS), thus promoting malignant transformation (Fig. 3b).^58,71,77^

Recently, the mechanism by which ∆Np63 maintains stem cell potential has been elucidated.^78^ ∆Np63 augments hexokinase-2 (HK-2) expression by binding to the p63-binding motif in the 15th intronic region of the HK-2 genomic sequence, which works as an enhancer.^78^ HK-2, via its mitochondrial-binding motif (MBF), affixes voltage-dependent anion channel 1 (VDAC-1) in the outer mitochondrial membrane (OMM, red).^78^ VDAC-1 in turn interacts with adenine nucleotide translocase-1 (ANT-1) located in the inner mitochondrial membrane (IMM, blue), forming a channel between IMM and OMM.^78,79^ This mechanism allows rapid ADP/ATP cycling through increased coupling between glycolysis and OXPHOS.^78^ Augmented coupling between glycolysis and OXPHOS protects cells from oxidative stress by reducing ROS formation^79^ and thereby preventing senescence.^80^ This allows for higher respiratory rates and increases the stem cell capacity (Fig. 3b).^78,79^

In addition, ∆Np63α-arbitrated lymphoid-specific helicase (Lsh) upregulation^77^ can epigenetically suppress fumarate hydratase (FH), a Krebs cycle enzyme.^81^ This leads to the accumulation of fumarate, considered an oncometabolite, which then inhibits the inhibitors of hypoxia-inducible factor (HIF), the prolyl hydroxylases (PHDs).^82^ Consequently, amplified HIF can promote malignancy through the Warburg effect by providing cancer cells with building blocks (Fig. 3b).^83^

∆Np63α has been shown to be reactivated in OED and OSCC.^14,78^ Upregulated ∆Np63 promotes oral cancer chemoresistance and proliferation by activating EGFR, multidrug resistance-associated protein 1 (MRP1)/ATP-binding cassette subfamily C member 1 (ABCC-1), ALDH1 and CD44 (Fig. 3b).^76^ A pervasive signalling network promoting stemness in oral cancer exists, involving ∆Np63α, hyaluronan synthase 3 (HAS-3), hyaluronic acid (HA), CD44, EGFR and signal transducer and activator of transcription-3 (STAT-3).^76^ ∆Np63α binds to the HAS-3 and CD44 promoter, upregulating their expression and promoting their translocation to the cell membrane.^76,84^ Membranous HAS-3 promotes the intracellular synthesis of HA from precursors uridine diphosphate N-acetylglucosamine (UDP-GlcNAc) and uridine diphosphate–glucuronic acid (UDP–GlcA), and then HA is extruded out of the cell membrane.^84^ The exteriorized HA then binds to its receptor CD44, which transactivates EGFR.^76^ Activated EGFR through STAT-3 upregulates ∆Np63α.^85^ ∆Np63α has STAT-3-binding elements in its promoter, fulfilling this purpose (Fig. 3b).^86^ This mechanism explains the attainment of stem cells in oral cancer via EGF. ∆Np63α indirectly upregulates ALDHA1 through the ∆Np63α/GSK-3/β-catenin (β-CAT)/ALDHA1 pathway (Fig. 3b and Supplementary Information).^62,63^

### c-MYC as a stem cell marker in OSF and its malignant evolution

Stromal hypoxia is an essential factor in the pathogenesis of OSF,^2,13,14^ and is mediated through several pathways.^2^ Hypoxia-induced enzymes, such as Cathepsin-D, H and E3 ubiquitin ligases such as F-box and WD-repeat domain containing 7 (FBXW-7) and DNA damage-binding protein 1 (DDB-1) can promote the proteolysis and proteasomal degradation, respectively, of the c-MYC protein.^87^ This can result in the loss of proliferative potential and epithelial atrophy. Similarly, the downregulation of c-MYC through hypoxia-induced HIF-2α can decrease stemness,^88^ leading to atrophy in OSF. As discussed previously, the atrophic epithelium in OSF shows low c-MYC expression (Fig. 1a).^13^

Several studies have shown c-MYC to be a necessary positive modulator of the proliferative compartment.^89,90^ The nuclear expression of c-MYC in the basal and parabasal cells of NOM ascribes its proliferative potential. The loss of nuclear c-MYC expression in the differentiated layers of oral mucosa further substantiates its role in maintaining basal stemness.^13^ c-MYC overexpression in tumours has been attributed to multiple mechanisms, including stabilizing mutations, amplifications and chromosomal translocations.^87^ In addition, it is upregulated by β1-integrin through the FAK/PI3K/AKT pathway (Fig. 2d),^69,70^ and by NF-κB through the Src/Rac and integrin-linked kinase (ILK)/AKT pathways.^69^ Interestingly, the NF-κB1/p50 subunit of NF-κB has been shown to inhibit c-MYC protein degradation via its inhibition by the E3 ubiquitin ligase FBXW-7,^91^ thus leading to elevated c-MYC levels (Fig. 1b).

Oct-4 and SOX2 are upregulated due to the downregulation of miR-145 by arecoline.^18^ As miR-145 is a positive regulator of E-cadherin and a negative regulator of Snail (SNAI1) and Slug (SNAI2), EMT occurs. The exposed epithelial cells thus acquire increased chemoresistance, augmented migration, increased invasiveness and anchorage-independent growth. Furthermore, SOX2 and Oct-4 expression is inversely related to miR-145 expression in the tissues of individuals with areca quid-induced OSCC.^18^ Since miR-145 downregulates c-MYC, the suppression of miR-145 drives the upregulation of c-MYC (Fig. 1b).^92^

### Bmi-1 is a stem cell marker in OSF and its malignant evolution

Bmi-1 is positively regulated by c-MYC and increases cellular proliferation by suppressing the INK4a locus (Fig. 2d).^93–95^ Downregulated Bmi-1 can propagate epithelial atrophy in OSF^71,77^ via enhanced senescence (Fig. 2c).^95^

The β1-integrin/FAK/PI3K/AKT/AP-1 pathway leads to the activation of Bmi-1^96^ through c-MYC (Fig. 2d).^95^ Whilst Bmi-1 expression is limited to the basal layer in normal epithelium, dysplastic and carcinomatous epithelium show Bmi-1 expression in the superficial layers.^95^

Importantly, the knockdown of Bmi-1 in normal epithelium did not cause an immediate arrest of replication or a loss of viable cells, whereas in OSCC cells, it had these two effects.^95^ In addition, the knockdown of Bmi-1 was shown to inhibit the tumour-initiating properties of ALDH1^+^ cells,^27,97^ enhance their radiosensitivity and^27,97^ chemosensitivity^27^ and inhibit metastasis.^97^ On the other hand, the overexpression of Bmi-1 alters ALDH1^−^ cells to become ALDH1^+^ cells, increasing tumour volume and enhancing metastatic foci.^97^ The coexpression of Bmi-1/Snail/ALDH1 correlates with the worst prognosis in oral cancer patients.^97^ Bmi-1 is thus considered to be a potential chemotherapeutic and radiotherapeutic target for increasing the sensitivity of CSCs.^27^

### Keratin 5/14 as a stem cell marker in OSF and its malignant evolution

#### Downregulated epithelial stemness via K-5/14 inhibition

Keratin 5/14 are normally expressed in basal-proliferating keratinocytes through activator protein 1/2 (AP-1/2) or specificity protein 1,3 (SP-1,3)^64^ along with ∆Np63. The expression of keratin 5/14 is mediated by the binding of ∆Np63 with AP-1,2 or SP-1,3 in the K-14 enhancer. In addition, in the K-14 promoter, ∆Np63 binds with AP2 to upregulate K-14 expression. In the K-5 promoter, ∆Np63 binds to upregulate its expression.^61,65^ The primary aetiological agent of OSF, ANE, ostensibly downregulates Keratin 5/14 through TGF-β1-mediated ∆Np63 abrogation,^61,65^ although it upregulates AP-1/2 and SP-1,3 (Fig. 4a).^64^

#### Regaining epithelial stemness through K-5/14 upregulation

EMT in OSF is mediated through the downregulation of membranous E-cadherin and β-CAT, as well as miR-205 and par-3 family cell polarity regulator (PARD-3), and the upregulation of N-cadherin, Twist-1, Zinc finger E-box-binding homeobox 1 (ZEB-1), MMP-9 and Vimentin, a mesenchymal-specific protein and a tell-tale marker of EMT in epithelial cancers.^12^ Subsequently, the expression of vimentin, which is associated with some degree of fibrosis in OSF,^98^ has been shown to upregulate keratin 5/14 through ∆Np63 in OSCCs.^65^ Even ∆Np63 itself promotes EMT,^12^ which is shown to promote stemness (discussed later). It has been established that a higher coexpression of vimentin and K-14 correlates with the recurrence and poor survival of OSCC (Fig. 4b).^65^

### Keratin-19 as a stem cell marker in OSF and its malignant evolution

Keratin-19 expression, restricted to epithelial cells, is essential for the maintenance of the proliferative potential of the basal stem cell layer in nonkeratinized mucosa. Its loss correlates with the loss of self-renewal capacity and subsequent atrophy in OSF.^8,99^ The atrophic epithelium further facilitates fibrosis through enhanced permeability.^8^ High-throughput oligonucleotide microarray analysis has demonstrated K-19 to be the topmost among 129 downregulated genes in OSF.^8^ Incidentally, K-19 expression shows an inverse correlation with arecoline concentration.^99^

Cytokeratin fragment antigen 21-1 (CYFRA21-1), a cytokeratin fragment produced through the action of caspase-3 on keratin-19, has been reported in the serum and saliva of patients with fibrosis, including OSF, as well as in those with malignancy.^100^ In vitro experiments have shown the cessation of CYFRA21-1 release into the culture supernatant with the addition of a caspase-3- specific inhibitor (Fig. 5a).^101^ Stromal hypoxia leading to caspase-3 and -9 activation is the most plausible explanation for the downregulation of K-19 and the appearance of CYFRA21-1 in OSF.^102,103^ CYFRA21-1 is thus a promising noninvasive proxy marker of hypoxia-initiated epithelial apoptosis and atrophy in OSF patients (Fig. 5a).^101^

While K-19 is downregulated in OSF, it is upregulated in OED.^100,104^ Furthermore, it demonstrates a sequential increase with progressive grades of OED and OSCC.^104,105^ Moreover, the expression of K-19 is the highest at the invasive front,^105^ and serves as an independent predictor of poor prognosis.^106^ Although the mechanism for alterations in K-19 expression in the malignant transformation of OSF has not been studied, increased K-19 expression in OED, OSCC and malignant OSF could be mediated through enhanced matrix stiffness, which itself is due to progressive matrix cross-linking. The increased stiffness in OSF mucosa is evident by a thickened basement membrane, subepithelial fibrosis and increased collagen density.^12^ The enhanced matrix stiffness increases myofibroblast formation, which in turn intensifies collagen formation.^2^ The increased collagen bioavailability and its binding to discoid domain receptor-2 (DDR-2) enhances constitutive src activation.^107^ The upregulation of K-19 is due to the phosphorylation of its tyrosine 394 residue by the constitutive activation of src kinase, which otherwise is not phosphorylated in the basal state (Fig. 5b).^108^ This will lead to a complex phenotype of increased K-19 breakdown evidenced by CYFRA21-1 expression in saliva/serum with a superimposed upregulation of K-19 through a previously discussed fibronectin- and/or type-1 collagen/β_1_-integrin/Src-based mechanism.

Increasing matrix stiffness through a progressively cross-linked ECM might mediate the upregulation of Yes-associated protein 1 (YAP-1) and its transcriptional coactivator with PDZ-binding motif (TAZ)^109–111^ in OSF. Interestingly, ∆Np63α forms a complex with YAP-1, which promotes stem cell survival.^112^ Moreover, YAP-1 can trigger SOX2 transcription by physical interaction with OCT-4.^113^ In addition, TAZ binds to the promoter of SOX2, which then drives stemness in oral cancer.^113^ YAP-1 upregulates CTGF (which induces proliferation) and c-MYC (which enhances clonogenicity), downregulates caspase-7 and BAX and upregulates Bcl-2 (which enhances apoptosis resistance) (Fig. 5b).^114^ These mechanisms might be relevant to the malignant transformation of OSF.

## Rise of cancer stem cells—dichotomy of areca nut ingredients in epithelial and stromal compartments

The origin of CSCs in OSCCs has been attributable^115^ to various factors, which are described as follows.

### The genetic alterations in the basal epithelial stem cells

It has been experimentally demonstrated that CD44^+^ is expressed in the basal cell compartment and absent in differentiated cells.^26^ The protracted contact of arecoline with oral keratinocytes leads to the upregulation of various stem cell markers, such as ALDH1, CD44, Nanog, Oct-4 and SOX2.^18^

### Upregulation of EMT in epithelial cells

The chronic use of areca nut leads to widespread effects, such as autophagy induction, promotion of EMT and increased stemness, which then promote oral cancer.^18^ CSCs are a plastic state of tumour cells undergoing EMT. Approximately 40–50% of oral cancer recurrence is attributed to EMT. Lazarevic et al.^39^ stated that EMT preferentially occurs at tumour margins, as they are sites of vessel invasion. The expression of EMT markers tended to be higher in surgical margins than within tumours.^39^

Oral cancer cells expressing SNAI1 (a master mediator of EMT) acquire a CSC-like phenotype, chemoresistance and migration and invasion potential.^45^ In cells expressing both ALDH1^+^ and CD44^+^, Snail coexpresses with ALDH1. Its experimental suppression decreases the expression of ALDH1, inhibits CSC-like properties and decreases tumorigenic potential. This suggests that in ALDH1^+^ cells, CSC properties are mediated by Snail, and that its reversal reduces chemoresistance.^43^

### Epithelial stemness augmentation by mesenchymal stem cells

Resident and bone marrow-derived mesenchymal stem cells (MSCs) are precursors of stroma associated with cancer.^29^ These stem cells contribute to angiogenesis and lymphangiogenesis, modulate the immune system and produce tumour-associated myofibroblasts.^29^ In OSCC, fibroblasts can be activated into myofibroblasts either through genetically transformed keratinocytes or exogenous agents such as irradiation or viruses.^28^ This in turn can stimulate the transformed keratinocytes by influencing stem cell division patterns towards symmetry, with an increase in the stem cell pool within the lesion.^28^

Via TGF-β, arecoline drives the upregulation of the mesenchymal stem cell (MSC) marker STRO-1 in OSF.^116^ The increased conversion of fibroblasts into myofibroblasts and upregulated stemness are indicated by their increased contraction, migration, invasion and expression of α-SMA and the pro α-1 chain of type-1 collagen.^116^ These MSCs can serve as a source of multipotent cells that can rapidly repopulate the wound in response to epithelial injury.^116^

Ye et al.^117^ showed that growth-regulated oncogene-α (GRO-α) secretion by OSF fibroblasts promotes oral keratinocyte malignant transformation by augmenting EGFR/ERK signalling, F-actin rearrangement and stemness. Transformed keratinocytes can then acquire further genetic alterations with the evolution of more invasive clones. Highly motile myofibroblasts may also come into close contact with the highly spindled transformed stem cells and fuse to produce a more aggressive cell with the myofibroblast property of high motility and the stem cell property of high self-renewal.^28^

Thus, there seems to be a dichotomy with respect to epithelial and mesenchymal clonogenicity in OSF in response to chronic areca nut chewing. Interestingly, the increased stemness of the stromal cell compartment drives a secondary increase in epithelial stemness, which could be an important mechanism of malignant transformation in OSF.

## Future perspectives

Various molecular pathways (Figs. 1–5) and pervasive epithelial–stromal interactions underlie the mechanisms of the upregulation of epithelial stem cells and their role in malignancy. Practically, the normal buccal mucosal epithelium, with an intact basal stem cell layer, has the potential to ameliorate fibrosis through the recompensation of basal stem activity. The restoration of the basal stem cell layer via stem cell therapy should be considered an important component of OSF therapy.^4,8^

Recent studies have demonstrated the importance of the perivascular stem cell niche in oral cancer. It is present in the ITF where CSCs reside, in close proximity to the blood vessels. Endothelial cell-initiated signalling has been shown to be critical for the survival and self-renewal of CSCs, and may play a role in resistance to therapy. Therefore, oral cancer patients might benefit from therapies that target CSCs directly or their supportive perivascular niche.^118,119^

Newly designed chemotherapy and radiotherapy regimens for the treatment of solid cancers have failed to improve patient survival. The underlying reason for this therapeutic failure is that these regimens target fast-dividing cells instead of slow-dividing CSCs.^120–123^ The slow cycling and constitutive expression of multiple members of the ATP-binding cassette (ABC) family of transporters is responsible for tumour stem cells exhibiting a high degree of chemoresistance.^124,125^ Thus, the final therapeutic goal should be to restore epithelial homoeostasis. Reinstating the renewal ability of the basal layer of oral mucosa should reverse fibrosis and prevent malignancy.^2,8,126–129^ Indeed, the coculture of normal buccal mucosal epithelial cell fibrotic fibroblasts causes the downregulation of connective tissue growth factor (CTGF),^128^ a profibrotic mediator in OSF.^2^ The undifferentiated keratinocytes reinstate the normal healing patterns in fibrosis through the downregulation of TGF-β and the stabilization of desmosomal assembly.^129^ Targeting the reactive tumour–stroma could be another approach to halt the signals from the microenvironment that prevent stem cell recovery.^28,31^

## Conclusion

There is compelling evidence to suggest that the aberrant self-renewing capacity of the basal stem cell layer in OSF with atrophic epithelium and its restitution in hyperplastic and/or dysplastic epithelium are associated with the progression to malignancy. Tumour–stroma interactions provide a niche to potentiate cancer stem cell behaviour, and the tumour-associated stroma has been shown to revive basal stem cell activity by initiating a vicious cycle between epithelial and stromal stem cell compartments.

It is possible that a reduction in stem cell activity causes epithelial atrophy in OSF, and its restitution promotes malignancy. The vacillating expression of keratin 5, 14, 19, CD44, β1-integrin, p63, Oct-4, c-MYC, c-MET and ALDH1 supports this hypothesis. There are no reports highlighting an oscillating relationship of OM-SCMs in atrophic OSF versus OPMDs and OSCCs. How the restitution of stem cell activity contributes to malignant transformation has been schematically illustrated for the first time. Furthermore, the role of OM-SCMs in atrophic OSF versus hyperplastic OSF, OED and OSCC is a fertile area of research, which may provide further credence in the treatment and management of OSF.

## Supplementary information

Supplementary Information (SI)
