# Supplementary material for: Loss of oral mucosal stem cell markers in oral submucous fibrosis and their reactivation in malignant transformation
Source: Int J Oral Sci. 2020 Aug 21;12:23. doi: 10.1038/s41368-020-00090-5 (PMC7442837; doi:10.1038/s41368-020-00090-5)
Supplement: Supplementary file 1 — Supplementary Information (SI) [file 41368_2020_90_MOESM1_ESM.docx]

Supplementary Information (SI)

Table: Oral Mucosal Stem Cell Markers and their Role in Mucosal Homeostasis

| **OM-SCM** | **Function** | **Comments** |
| --- | --- | --- |
| ***Keratin 5*** | Structural intermediate filament protein near ubiquitously expressed in all basal cells.[^22^](#_ENREF_22) | Expressed in the basal stem cell layer.[^22^](#_ENREF_22) K-5 promoter have a ∆Np63 (Another basal Stem cell Marker) binding site.[^61^](#_ENREF_61)^,^ [^64^](#_ENREF_64)^,^ [^65^](#_ENREF_65) Tongue epithelial cells co-expressing K-5 and β1-integrin (Stem Cell marker) form oral epithelial equivalents (OEE) in cell culture.[^22^](#_ENREF_22) |
| ***Keratin 14*** | Type I cytokeratin and intermediate filament protein.[^22^](#_ENREF_22) | Expressed in the basal stem cell layer.[^22^](#_ENREF_22) K-14 promoter and enhancer have a ∆Np63 (Another basal Stem cell Marker) binding sites.[^61^](#_ENREF_61)^,^ [^64^](#_ENREF_64)^,^ [^65^](#_ENREF_65) |
| ***Keratin 15*** | Type I cytokeratin and intermediate filament protein.[^22^](#_ENREF_22) | Restricted to basal stem cell layer.[^55^](#_ENREF_55)^,^ [^59^](#_ENREF_59) Although, it may be re-expressed on terminal differentiation, this follows the downregulation of β_1_-integrin (Stem Cell marker) and expression of differentiation specific Protein kinase C (PKC)/ Activator protein 1 *(*AP-1*)* pathway.[^59^](#_ENREF_59) The K-15 promoter contain Forkhead Box M1 (FOXM1) binding motif which mediates the basal stemness.[^59^](#_ENREF_59) |
| ***Keratin 19*** | Type I cytokeratin which is not paired with a basic cytokeratin | α_6_β_4_-integrin^+^CD71^-^ keratinocytes express Keratin 19 endorsing its role as stem cell marker.[^55^](#_ENREF_55) Restricted to basal layer especially in non-keratinized mucosa.[^8^](#_ENREF_8)^,^ [^55^](#_ENREF_55) |
| ***α_6_β_4_-integrin^+^CD71^-^*** | Expression is restricted to basal cell layer; it is essential for hemi-desmosomal assembly and binds to laminin-5 in basement membrane.[^22^](#_ENREF_22) | α_6_β_4_-integrin^-^ basal keratinocytes form terminally differentiated cells.[^55^](#_ENREF_55) The α_6_β_4_-integrin^+^CD71^-^ keratinocytes display more stemness when compared to α_6_β_4_-integrin^+^CD71^+^ cells.[^55^](#_ENREF_55) These cell are also positive for other stem cell markers Oct3/4, CD44H, Keratin-19 and p63.[^55^](#_ENREF_55) |
| ***β_1_-integrin*** | Constituent of integrin complex that binds to basement membrane molecules.[^22^](#_ENREF_22) | Basal keratinocytes show highest expression, while those committed to differentiation show decreased expression of β1-integrin.[^22^](#_ENREF_22)^,^ [^55^](#_ENREF_55) |
| ***Collagen IV*** | Chiefly located in basement membrane.[^22^](#_ENREF_22) | Enrich basal stem cell layer by allowing adhesion of basal stem cells to the basement membrane.[^22^](#_ENREF_22) |
| ***p75^NGFR^*** | Low affinity receptor that binds to nerve growth factor.[^22^](#_ENREF_22)^,^ [^60^](#_ENREF_60) | Protects basal stem cells by endowing them with apoptosis resistance.[^22^](#_ENREF_22) p75^NGFR+^ keratinocytes are able regenerate buccal mucosal epithelium in a wound healing model.[^60^](#_ENREF_60) Present only at the tips of connective tissue papillae and rete ridges, indicating it as a specific OM-SCM.[^22^](#_ENREF_22) |
| ***SSEA1*** | Cell surface protein involved in cellular differentiation | The spheroids from oral mucosa derived stem cell expressed SSEA1.[^56^](#_ENREF_56) |
| ***CD24*** | Signal transducer | Utility in isolating OM-SC is not proven.[^55^](#_ENREF_55) |
| ***CD44H*** | Hyaluronan Receptor,[^76^](#_ENREF_76) the smallest variant is called Standard CD44 (CD44s)/Hematopoietic-CD44 (CD44H). | Cell adhesion molecule (CAM) associated with stemness; expression is detected in basal stem cell layer not in differentiated cells.[^22^](#_ENREF_22) |
| ***CD71*** | Serves as a Transferrin receptor[^22^](#_ENREF_22) | Negative Marker, found in lower level in stem cells.[^60^](#_ENREF_60) |
| ***CD117 (c-kit)*** | Cytokine growth factor stem cell receptor | Negative Marker, found in lower level in stem cells.[^22^](#_ENREF_22) |
| **CD133** | Function not clearly known | Expressed in basal layer of neonatal epidermis, its loss is followed by appearance of differentiation marker involucrin.[^55^](#_ENREF_55) Its utility in isolating OM-SC is not proven.[^55^](#_ENREF_55) |
| ***MCSP*** | Involved in migration and invasion of melanoma cells. | Promote stem cell clustering through augmented cell-cell adhesion.[^22^](#_ENREF_22) |
| **Nestin** | Intermediate filaments in neural stem cells | The spheroids from oral mucosa derived stem cell expressed higher Nestin when compared to those from skin.[^56^](#_ENREF_56) |
| **p63/∆Np63α** | Essential for epithelial homeostasis.[^58^](#_ENREF_58) | The lose designation of p63[^55^](#_ENREF_55) should be avoided as only ∆Np63α isoform dictates basal stemness.[^61^](#_ENREF_61) ∆Np63α binds to promoters of p16INK4a and p19ARF repressing their expression and averts cellular senescence.[^58^](#_ENREF_58) |
| ***Oct-3/4*** | Embryonic Stem cell marker. | The spheroids from oral mucosa derived stem cell expressed higher expression Oct-3/4.[^56^](#_ENREF_56) |
| ***Nanog*** | Homeobox protein in embryonic stem cells, helping them to maintain pluripotency. | The spheroids from oral mucosa derived stem cell expressed Nanog.[^56^](#_ENREF_56) |
| **SOX2** | Transcription factor containing High Mobility Group box (HMG-box) protein domains.[^22^](#_ENREF_22) | The spheroids from oral mucosa derived stem cell expressed higher expression of SOX2 when compared to those from skin.[^56^](#_ENREF_56) |
| ***ABCG-2*** | Protects cell against toxic chemicals by transporting them out of the cells.[^22^](#_ENREF_22) | Stem cell marker in several different tissues.[^22^](#_ENREF_22) |
| ***ALDH-1*** | Cellular detoxification of ROS.[^63^](#_ENREF_63) | Evidence for it being an OM-SCM is indirect as ∆Np63α inhibits Glycogen synthase kinase 3 β (GSK-3), which in turn inhibits beta-catenin (β-CAT) leading to β-CAT nuclear accumulation.[^62^](#_ENREF_62) β-CAT in once nucleus binds to promoter of ALDHA1 to upregulate its expression.[^63^](#_ENREF_63) |
| ***Bmi-1*** | Relevant to renewal of hematopoietic and neural stem cells.[^57^](#_ENREF_57) | Identified as OM-SCM though in-vivo lineage tracing.[^22^](#_ENREF_22) Bmi-1 is a marker of lingual epithelial stem cells along with K-5 and K-14.[^57^](#_ENREF_57) Shown to be important for rapid regeneration of lingual epithelium post injury.[^57^](#_ENREF_57) |
| **Connexin-43 (Cx43)** | Gap junction protein Cx43 is essential for stratification of oral mucosa.[^55^](#_ENREF_55) | Negative marker as its downregulation in oral keratinocytes followed by high levels of OM-SCMs p63 and β_1_-integrin.[^55^](#_ENREF_55) |
| **Desmoglein-3 (Dsg-3)** | Cell adhesion molecule | Negative marker of Stem cells.[^55^](#_ENREF_55) |
